# HELP-DKT: an interpretable cognitive model of how students learn programming based on deep knowledge tracing

**DOI:** 10.1038/s41598-022-07956-0

**Published:** 2022-03-07

**Authors:** Yu Liang, Tianhao Peng, Yanjun Pu, Wenjun Wu

**Affiliations:** 1 School of Computer Science and Engineering, Beihang University, Beijing, 100091 China; 2 Shen Yuan Honors College, Beihang University, Beijing, 100091 China

**Keywords:** Computer science, Software, Mathematics and computing, Information technology

## Abstract

Student cognitive models are playing an essential role in intelligent online tutoring for programming courses. These models capture students’ learning interactions and store them in the form of a set of binary responses, thereby failing to utilize rich educational information in the learning process. Moreover, the recent development of these models has been focused on improving the prediction performance and tended to adopt deep neural networks in building the end-to-end prediction frameworks. Although this approach can provide an improved prediction performance, it may also cause difficulties in interpreting the student’s learning status, which is crucial for providing personalized educational feedback. To address this problem, this paper provides an interpretable cognitive model named HELP-DKT, which can infer how students learn programming based on deep knowledge tracing. HELP-DKT has two major advantages. First, it implements a feature-rich input layer, where the raw codes of students are encoded to vector representations, and the error classifications as concept indicators are incorporated. Second, it can infer meaningful estimation of student abilities while reliably predicting future performance. The experiments confirm that HELP-DKT can achieve good prediction performance and present reasonable interpretability of student skills improvement. In practice, HELP-DKT can personalize the learning experience of novice learners.

## Introduction

Currently, an increasing number of novice students choose to learn programming online, especially via Massive Open Online Courses (MOOCs). In such a scenario, a crucial problem is how to facilitate students to learn programming (HELP) via intelligent tutoring. In this paper, this problem is denoted as the HELP problem.

A programming language can be decomposed into a list of concepts, which can be recognized as knowledge components (KCs). Every programming exercise involves multiple concepts or KCs, which can be represented in the form of a Q-matrix^1^. To address the HELP problem, it is needed to build an interpretable cognitive model that can determine students’ mastery level of KCs by mining their learning trajectories in programming exercises. A knowledge tracing (KT) model^2^ is commonly adopted in the online-teaching domain because it can predict the probability of answering the next exercise correctly. Recently, the deep knowledge tracing (DKT)^3,4^, where a deep neural network-based cognitive model is used for learning how to program, has been proposed. Despite its good prediction performance, this approach can cause difficulties in interpreting the students’ learning status on each of the programming concepts. Specifically, there have been two major factors contributing to the interpretation problem.

First, the current implementation of the DKT-based model for programming courses captures student’s learning interactions through programming exercises and saves them in the form of a set of binary responses. Such binary sequences merely indicate whether a student’s code could run the test cases of an exercise correctly or not but fail to utilize rich features of source codes in the learning process. However, these features of each run of a student’s program may include the success number of test cases, the source code, and all errors in the source code. The feature-rich information enables extending the original DKT model to improve both prediction and interpretability performances. For instance, the abstract syntax tree (AST) of a source code can be transformed into the input vectors of the DKT model by encoding the source code to a vector representation via embedding in the natural language processing (NLP)^5^.

Second, the black-box nature of the DKT model makes it difficult to present explainable prediction results to instructors of programming courses. An instructor not only concerns about whether students can successfully finish their homework passing all test cases for an exercise but also wants to know about every student’s ability level on each of the programming concepts. For instance, when a student’s code fails to pass test cases, the instructor would like to check the errors in the student’s code and identify his understanding level of certain particular concepts, e.g., strings or conditionals. In this way, the instructor could diagnose the student’s weakness in mastering the programming concepts and provide personalized teaching strategies for him.

In order to solve the HELP problem, this paper proposes a HELP-DKT model that aims at incorporating feature-rich input vectors and providing personalized conceptual-level skill assessments for students. In the proposed model, student’s conceptual skills in programming are represented in the form of a Q-matrix, and the corresponding cognitive elements are designed as an extra layer over the DKT model. This structural change in the HELP-DKT model design improves both predictive accuracy and interpretability. By precisely identifying programming errors in every student’s code, the HELP-DKT model can infer students’ skills on each of the concepts and track temporal skill changes over the sequence of code submissions. The experimental results confirm that the proposed HELP-DKT model has excellent performance and visualization ability in displaying dynamic changes in students’ skills.

The main contributions of this work can be summarized as follows:

- A program embedding is proposed for encoding source codes to vector representations, and error classifications are incorporated as concept indicators into a personalized Q-matrix. Using rich-feature input vectors, the HELP-DKT model can describe learning trajectories of students in a fine-grained way, achieving highly accurate predictive performance.
- An extra cognitive layer is introduced in the DKT framework to create a fully-connected interaction between the hidden skill state of the DKT and the personalized Q-matrix. Therefore, the HELP-DKT is capable of inferring student abilities on the conceptual level and presenting visualized interpretations of dynamic change in students’ skills to course instructors.
- To facilitate further research, the code and relevant dataset have been published in the following URL: https://github.com/liangyubuaa/HELP-DKT. A detailed description about how to use the code and dataset is provided in this page.

The rest of the paper is organized as follows. An overview of the related work is presented in “Related work” section. In “Methods and experiments” section, the details of the proposed methods are given, and the implementations and experiments of the proposed model are provided. In “Results and discussion” section, the results of the experiment are described and discussed. Lastly, the conclusions and future work directions are given in “Conclusions” section.

## Related work

### Student cognitive model

In an intelligent tutoring system for programming courses, a student cognitive model has been often needed to describe students’ cognitive states during their studying. Early research efforts in this field highlighted observable gaps between students’ understanding of core programming concepts and their capability of applying these concepts to the construction of simple programs^6^. Therefore, modeling the learning process of novice students in programming courses involves describing the temporal development of multiple latent cognitive skills.

Prior research efforts have mostly adopted Bayesian knowledge tracing (BKT) models, item response theory (IRT) based models or some other user behavior analysis models to build student models. Papers^7,8^ are based on gated recurrent unit (GRU) model while papers^9,10^ are focus on solving the link prediction task. These work propose good prediction models. However, a limitation with these work is that they do not fully leverage the students’ historical attempt dataset.

The Bayesian knowledge tracing (BKT)^2^ provides an effective way to model temporal development of cognitive skills using the Bayesian inference with a hidden Markov model. However, the conventional BKT model-based approach^11^ is not suitable for programming courses because it does not support a multi-dimensional skill model and requires additional algorithms to create a Q-matrix.

Some of the related studies adopted the IRT extensions for student’s skills modeling in programming courses. Yudelson et al.^12^ used a variant additive factors model (AFM) to infer students’ knowledge states when solving Java programming exercises. Rivers et al.^13^ analyzed the students’ Python programming data by fitting learning curves using the AFM to identify which programming concepts were the most challenging for students to master the Python programming. The advantage of the mentioned AFM-based methods over the BKT-based methods is their capability to tackle scenarios of multi-dimensional skills. However, both mentioned methods regard students’ programming trajectories as sequences of binary responses while ignoring rich features embedded in different versions of students’ codes during the submission attempts.

Our previous work^14^ aimed to address the above-mentioned issue and adopted the conjunctive factor model (CFM)^15^ to establish a better cognitive relationship based on students’ learning data. The core concept of the CFM is a boolean Q-matrix, which is a pre-required matrix for describing the relationship between items and skills. The limitation of the CFM is that it does not treat multiple skills in one item differently, which might lead to inaccurate skill assessment. The CFM was extended to the personalized factor model(PFM) by using programming error classification as a cognitive skill representation. By introducing this modification, the predictive performance of the CFM for learning to program has been significantly improved. Both CFM and PFM are shallow model, and their main limitation is that they cannot handle large datasets.

Recently, a number of deep neural network-based KT models have been proposed. The Deep-IRT^16^ is an extended DKT model, which has been inspired by Bayesian deep learning. The Deep-IRT can achieve better prediction performance than shallow structured models, but it lacks personalized descriptions of students in the input layer due to fixed, binary Q-matrix designed by experts. In online program teaching, Wang et al.^4^ used a recurrent neural network (RNN) and focused on students’ sequences of submissions within a single programming exercise to predict future performance. The main shortcoming of the DKT model is poor interpretability caused by the black-box nature of a deep neural network. Also, it does not specify the probabilistic relationship between latent skills and student codes in the form of a Q-matrix, which makes it hard for instructors to understand the analysis results of the DKT.

### Program vector embeddings

Methods for vectorizing programs have many similarities with the representation learning methods, such as the vector embedding technique presented in^5^. In the program analysis domain, Piech et al.^17^ introduced a neural network method, which encoded programs as a linear mapping from an embedded precondition space to an embedded postcondition space. Peng et al.^18^ proposed a novel “coding criterion” to build vector representations of nodes in ASTs, which have provided great progress in program analysis. BigCode^19^ is a tool that can learn AST representations of given source codes with the help of the Skip-gram model^20^.

The above-mentioned methods have achieved good results, which has enlightened us to make the best use of vector embeddings that include rich information. This approach offers the possibility of using program codes as the input of deep learning models, especially student cognitive models.

### Automated program repair

In online programming education, many tools have been adopted to repair student error codes automatically. These tools are collectively referred to as automated program repair (APR) tools. For instance, Qlose^21^ is an approach used to repair students’ programming attempts in the education field automatically. This approach is based on different program distances. The AutoGrader^22^ is a tool that aims to find a series of minimal corrections for incorrect programs based on the program synthesis analysis. This tool requires course teachers to provide basic materials, such as a list of potential corrections based on known expression rewrite rules and a series of possible solutions for a certain problem. Gulwani et al.^23^ proposed a novel APR technique for introductory programming assignments. The authors used the existing correct students’ solutions to fix the new incorrect attempts. A limitation of this solution is that it cannot provide educational feedback to students and instructors.

The above-presented tools aim at fixing the wrong codes or getting the right repair results, but they neither examine the error types of students in detail nor try to integrate the outputs with the student cognitive model. However, these error types contain rich information that reflects the student’s weakness, which is very useful in the intelligent tutoring field.

## Methods and experiments

### Program vector embeddings

Creating vector embeddings for student codes is necessary to incorporate features of source codes into the DKT model. These vector embeddings represent the characteristics and structural features of students’ code submissions of programming exercises. This paper presents a three-step method for program vector embeddings, inspired by NLP^5^ domain.

*Step 1* The first step in code vectorization is to gain an abstract syntax tree (AST) from a source code. The AST is a compressed tree for representing a program structurally. In an AST of a program, a node (e.g., variable, constant, and statement) corresponds to a program component. Thus, an AST can capture the entire structural information of a program and can be mapped back into it. Furthermore, because of the finite number of types and nodes in an AST, it can be vectorized.

*Step 2* The second step is to generate *node* vectors in ASTs. In this step, each node in ASTs is trained and map to a real-valued vector, which contains each feature of the node. Inspired by BigCode tools^19^, the Skip-gram model^20^ is used to compute *node* vectors. The principle of this model is to use the currently known nodes to predict the context of them. Finally, the skip-gram model outputs a Huffman tree, where each leaf node represents a certain program component.

*Step 3* The final step is to generate the whole *program* vector assembled by *node* vectors. Compared to the NLP domain, the *node* vector is analogous to the *word* vector while the *program* vector is similar to the *sentence* vector. In the NLP domain, common strategies of learning sentence representations are to compute the average or the weighted average of pre-trained *word* vectors (e.g., word2vec, TF-IDF). On the basis of these strategies, a new method is proposed to update the vector representation of each *node* recursively based on the structural and frequency information of that node and its direct children in the AST. Particularly, the updating process of a *node* vector is given by Eqs. (1–3). It should be noted that the updating process is executed from bottom to top, where the vector representation of the root *node* is regarded as a vector representation of the whole program.

1 $$\begin{aligned} \mathbf{p} _n&=\mathbf{vec _n} \cdot e^{td_n} \end{aligned}$$

2 $$\begin{aligned} \mathbf{c} _n&=\sum _{i=1}^m\frac{cnt_{n_i}}{cnt_n} \cdot \mathbf{vec _{n_i}} \end{aligned}$$

3 $$\begin{aligned} \mathbf{vec _n}'&=tanh\left( \frac{1}{m+1}{} \mathbf{p} _n + \frac{m}{m+1}{} \mathbf{c} _n\right) \end{aligned}$$

In Eqs. (1–3), $\mathbf{vec} _n$ denotes the original vector representation of a node *n* in an AST $\mathcal {A}$; node ${n_1 , n_2,\ldots , n_m}$ are *m* direct children of the node *n* and $\mathbf{vec _{n_1}, \mathbf{vec} _{n_2},\ldots , \mathbf{vec} _{n_m}}$ are the corresponding original vector representations, which are calculated in Step 2; ${cnt_n}$ denotes the total number of nodes under *n* in $\mathcal {A}$ (i.e., the position information of *n* in $\mathcal {A}$); ${td_n}$ is the TF-IDF value of *n*, reflecting its frequency information in the AST; $\mathbf{p} _n$ stands for the comprehensive information of *n* multiplied by its vector representation and TF-IDF value (Eq. 1); $\mathbf{c} _n$ indicates the information of *m* direct children of *n*, which represents the sum of the weighted vectors (Eq. 2). The weights (${\frac{cnt_{n_i}}{cnt_n}}$) are weighted by the number of nodes under ${n_i}$; $\mathbf{vec _n}'$ is the updated vector representation of the node *n*, which represents the weighted average of the comprehensive information of node *n* ($\mathbf{p} _n$) and its *m* children nodes ($\mathbf{c} _n$) (Eq. 3). The weights are set to ${\frac{1}{m+1}}$ and ${{\frac{m}{m+1}}}$, and function $tanh(\cdot )$ is used to normalize the result.

### Personalized Q-matrix

To represent the conceptual skills in the HELP-DKT model, a Q-matrix is used to describe the relationship between the programming concepts in the form of KCs and every programming exercise^1^. In the Q-matrix, a cell value of one at row *i* and column *j* indicates that exercise *i* involves concept *j*; otherwise, it is set to zero. The definition of the Q-matrix enables to distinguish different exercises.

To distinguish different students for the purpose of personalization, the details of student codes are determined. An effective APR tool similar to the one described in^14^ is proposed. Using this tool, wrong student codes can be fixed, and the correct repair results can be obtained. The APR tool mainly includes two steps, which are as follows. In the first step, for a given programming assignment, the tool can automatically cluster the AST form of the correct student codes using dynamic program analysis. In each cluster, one of the grouped codes is randomly selected as a specification. In this way, all specifications can be seen as a solution space of the assignment. In the second step, given a wrong student attempt, the tool starts to run a repair procedure on the student submission against all source code specifications and automatically finds the optimal match in the solution space. Thus, the proposed tool can generate minimal repair patches for a wrong attempt and identify the corresponding error types. Moreover, this tool can accurately associate error types with the major concepts of a programming language (e.g., Python). Finally, the error types show the student’s misunderstanding level of certain programming concepts or low cognitive skills of applying these concepts to constructing the program components. Based on the feature-rich output of the APR tool, a personalized Q-matrix denoted as P-matrix in the following is constructed. The same as for a Q-matrix, rows of P-matrix stands for a student’s attempts to solve exercises while columns represent the programming concepts. Specifically, a cell value of one in row *i* and column *j* suggests that not only attempt *i* involves concept *j* but also a student has applied *j* correctly. A cell value of zero means the opposite. In this way, the P-matrix associates exercises, concepts, and students, thus achieving the property of personalization. Clearly, an attempt is successful if and only if the corresponding P-matrix’s row equals the relevant Q-matrix’s row; that is, the student has mastered all the concepts involved in the exercise.

**Figure 1:**
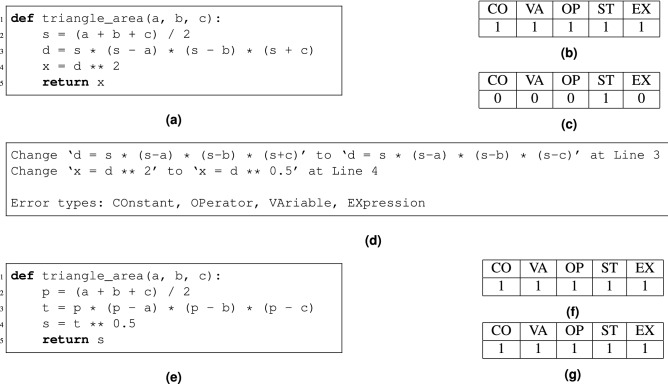
The illustration of buggy and correct attempts and the corresponding rows in the Q and P matrices. (**a**) Buggy attempt **B1**. (**b**) Row for **B1** in Q-matrix. (**c**) Row for **B1** in P-matrix. (**d**) Repair result for **B1**. (**e**) Correct attempt **C1**. (**f**) Row for **C1** in Q-matrix. (**g**) Row for **C1** in P-matrix.

To explain the relationship between attempts and their relevant Q/P-matrix better, an example is given in Fig. 1. Figure 1a shows a buggy attempt **B1**, and Fig. 1e displays the correct attempt **C1**; both attempts relate to the same program problem, which is in this case calculating the area of a triangle. This problem involves five major concepts, which are constants (co), variables (va), operators (op), strings (st), and expressions (ex). Thus, by definition of the Q-matrix, the rows corresponding to **B1** and **C1** in the Q-matrix are the same, as shown in Fig. 1b,f. After repairing **B1** using the proposed APR tool, the repair results shown in Fig. 1d are obtained. The result shows that the APR tool can accurately identify the error types of **B1**. Based on the repair result, the row corresponding to **B1** in the P-matrix can be obtained, as shown in Fig. 1c. However, **C1** includes no error, so the corresponding row in the P-matrix is the same as that in the Q-matrix, as shown in Fig. 1g.

### HELP-DKT framework

**Figure 2:**
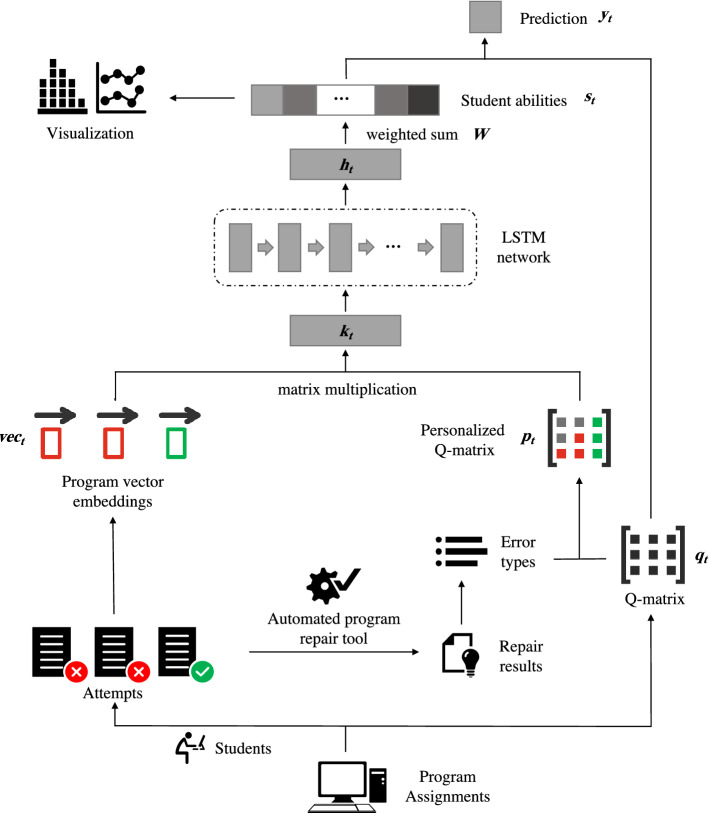
Framework of the HELP-DKT model.

The DKT is extended by introducing the *program vector embeddings* and *P-matrix* into the DKT and combining them as feature-rich student’s historical interactions. Besides, an extra cognitive layer is added to the DKT structure to obtain students’ ability levels. The framework of the HELP-DKT model is presented in Fig. 2, where it can be seen that it involves four major parts: integrating program vector embeddings and P-matrix as the input layer, tracking students’ latent cognitive status by an LSTM network, determining students’ mastery levels, and making a prediction. The HELP-DKT first receives a sequence of student’s interactions and then predicts the probability of answering the next exercise correctly and finally presents the student ability on each concept over time. To explain the HELP-DKT model better, a pseudo code is given in Algorithm 1.

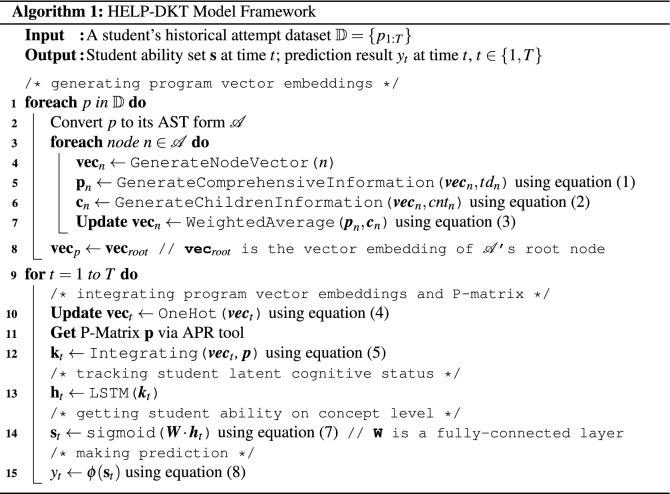



#### Integrating program vector embeddings and P-matrix

As mentioned previously, the program vector embeddings and P-matrix contain rich information about student’s submissions. Therefore, the program vector embeddings and P-matrix are integrated as the input layer of the HELP-DKT model to encode students’ abilities on the conceptual level and achieve a better prediction performance. The input layer is organized as follows:

4 $$\begin{aligned} \mathbf{vec} _t&=\left\{ \begin{aligned}&[v_1,v_2,\ldots ,v_{n},0,0,\ldots ,0], \quad \text {if correct}&\\&[0,0,\ldots ,0,v_1,v_ 2,\ldots ,v_{n}], \quad \text {if wrong}&\end{aligned} \right. \end{aligned}$$

5 $$\begin{aligned} \mathbf{k} _t&= \mathbf{vec} _t \cdot \mathbf{p} _t \end{aligned}$$

First, considering that the answer has a certain influence on the change in the student ability level, the *n*-dimensional one-hot encoding program vector is extended to the 2*n*-dimensional vector in order to distinguish the correct vector from the wrong one, as given in Eq. (4). Second, the extended code vector $\mathbf{vec} _t$ is multiplied with its corresponding P-matrix $\mathbf{p} _t$ at time *t* and used as the input $\mathbf{k} _t$ of the LSTM network, as given in Eq. (5); $\mathbf{k} _t$ contains both the vector embeddings and features related to the programming exercises, students, and concepts.

#### Tracking student latent cognitive status by LSTM

An LSTM denotes a special RNN, which can learn long-term dependencies over students’ learning trajectories. The core of the LSTM is the cell state, which flows through the entire network. The LSTM can add and remove data from the cell state controlled by using the gate structure, which is designed to protect and control the cell state and information flow. Thus, the hidden state ($\mathbf{h} _t$) can be obtained, and it is determined by the cell state ($\mathbf{c} _t$) and the output gate ($\mathbf{o} _t$, which can be expressed as:

6 $$\begin{aligned} \mathbf{h} _t = f(\mathbf{o} _t,\mathbf{c} _t) \end{aligned}$$

In the proposed model, the gates of the LSTM are used to simulate student’s learning and forgetting processes. This structure can track students’ latent cognitive status from the hidden state $\mathbf{h} _t$ of the LSTM network, which represents the output of the cell state.

#### Getting student ability on conceptual level

Due to the black-box nature of the LSTM model and a lack of regularization of inherent learning cognitive constraints, the hidden state $\mathbf{h} _t$ in the DKT cannot accurately represent the temporal change of student’s skill levels in the process of improving the source codes. To present interpretation of skill dynamics better and retain high prediction performance simultaneously, the DKT is extended by introducing an extra cognitive mapping in the form of a fully-connected layer to output the student ability levels $\mathbf{s} _t$ explicitly, which can be expressed as follows:

7 $$\begin{aligned} \mathbf{s} _t = sigmoid(\mathbf{W} \cdot \mathbf{h} _t) \end{aligned}$$

First, because the dimension of $\mathbf{h} _t$ is determined by data and training goals of the LSTM, the fully-connected layer $\mathbf{W}$ is used to resize $\mathbf{h} _t$ so that the dimension of $\mathbf{s} _t$ can equal the total number of programming concepts. In this way, each element $s_{tj}$ of $\mathbf{s} _t$ corresponds to concept *j*. The *sigmoid* function is used as an activation function of the fully-connected layer to scale each $s_{tj}$ in the range of (0,1) and to infer the student’s ability level on concept *j* at time step *t*.

#### Making prediction

Based on the student’s ability level $\mathbf{s} _t$, the HELP-DKT can compute the probability $y_t$ that a student completes the exercise correctly at time step *t* as follows:

8 $$\begin{aligned} y_t = \varvec{\phi }(\mathbf{y} _t) = \varvec{\phi } \Big (sigmoid\big (\alpha \cdot (\mathbf{s} _t \otimes \mathbf{q} _t - \theta )\big ) \Big ) \end{aligned}$$

where $\otimes$ represents the mask operation, and $\varvec{\phi }$ represents multiplication of each element of a vector; the factor $\varvec{\theta }$ indicates the difficulty level of a concept, and $\varvec{\theta }$ is post labeled by domain experts, who may find different knowledge components of varying difficulty after reviewing the students’ submissions. First, $\varvec{\theta }$ is subtracted from $S_{t}$ to obtain the difference between the student’s mastery level and the concept’s difficulty level. If this difference is positive, it is considered that the student is capable of applying this concept correctly. Then, the mask operation is used to select all concepts involved in the exercise according to the corresponding Q-matrix ($\mathbf{q} _t$) so as to avoid the influence of concepts unrelated to the exercise. For instance, assume $\mathbf{s} _t=[s_{1},s_{2},s_{3},s_{4}]$,, and $\mathbf{q} _t=[1,0,1,0]$; then, the masking result $\mathbf{w} _t=(\mathbf{s} _t - \theta ) \otimes \mathbf{q} _t$ is $[s_{1}-{\theta }_1,s_{3}-{\theta }_3]$. The factor of $\alpha$ is set to 10.0 for a practical reason so that the maximum prediction result for a particular problem is close to 1.0, which means that the student has mastered the knowledge component. For instance, if the student ability is not scaled, the maximum value that can be obtained is $sigmoid (1-0.5) = sigmoid (0.5) = 0.62$. And when $\alpha$ is used, the maximum value that can be obtained is $sigmoid (10.0*(1-0.5)) = sigmoid (5) = 0.99$. After processing by the *sigmoid* activation function, each element of $\mathbf{y} _t$ can be computed as a probability that the student can apply a concept correctly at time *t*. Finally, based on the assumption that the probability $y_t$ of an attempt success depends on the probability of mastering all concepts associated with the particular programming exercise, each element of $\mathbf{y} _t$ is multiplied to generate the prediction result $y_t$.

### Dataset

The following experiments are designed to evaluate the HELP-DKT model. The experimental data were collected from a Python Programming Introductory course hosted on a MOOC platform (https://www.educoder.net) intended for learning a variety of programming languages. The dataset includes 9,119 source codes completed by novice students in six programming assignments. These assignments are arranged as step-by-step challenges for students. All challenges are designed based on ten basic Python concepts, which are: constants (co), variables (va), operators (op), strings (st), expressions (ex), lists (li), tuples (tu), dictionaries (di), conditionals (cd), and input/output (io). The difficulty levels of these concepts are marked by the field experts. The values of co, va and op are set to 0.3, the values of st, ex, li and tu are set to 0.4, and the values of di, cd and io are set to 0.5. It should be noted that students are allowed to submit multiple attempts for each challenge. Therefore, the dataset contains multiple intermediate versions of code submissions made by a student for each challenge, which can be used to infer the cognitive process of mastering the key programming concepts. The overview of the dataset is described in Table 1.

**Table 1:** Dataset overview.

Challenge	Topic	# Students	# Programs	# Correct	# Incorrect	Concepts
C-1	String concatenation	608	1038	591	447	VA, OP, ST, EX, IO
C-2	Modifying a list	553	2188	553	1635	CO, VA, OP, EX, LI, IO
C-3	Calculating quantities	452	788	446	342	CO, VA, OP, EX, IO
C-4	Sorting elements	312	1977	312	1665	VA, EX, TU, IO
C-5	Computing factorials	188	2236	188	2048	CO, VA, OP, ST, EX, CD, IO
C-6	Modifying a dictionary	72	892	72	820	CO, VA, ST, EX, DI, IO

### Experiments

#### Generating program embeddings

Using the proposed method, vector embeddings of all programs in the dataset can be easily generated. First, the source codes are encoded to a 10-dimensional vector representation, and then the experiment is conducted to verify the effectiveness of the obtained program vectors.

As mentioned above, the program vector represents structural information of source codes. Therefore, for the codes of the same challenge, the structures are similar, and the corresponding vector embeddings should also be similar. On the contrary, the code vectors of different challenges should show apparent differences. All vectors are categorized into six clusters that correspond to six challenges in the dataset, and then a 2D visualization of the result is generated.

**Figure 3:**
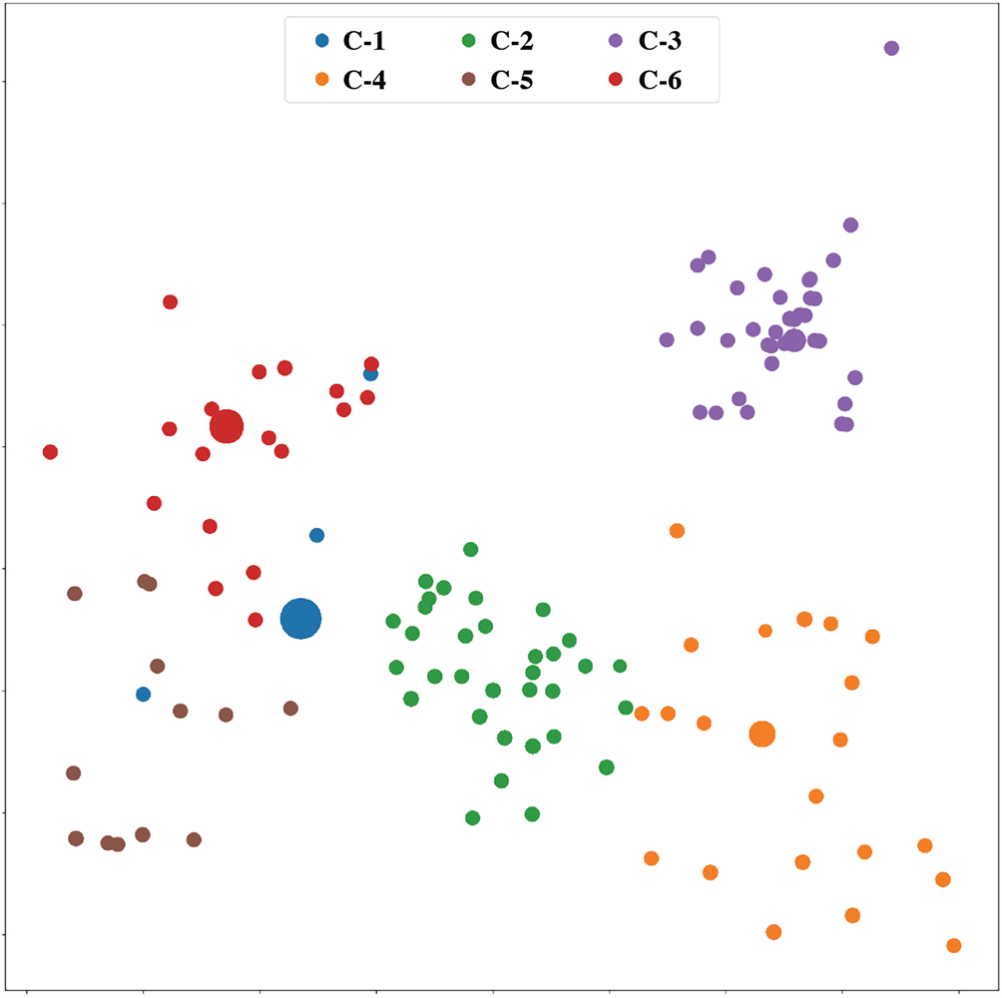
The clustering result of program embeddings. Each dot represents a program vector. The size of dots is proportional to the number of programs included in the same vector embeddings; six colors represent six challenges. Programs of the same challenge are grouped into the same category, while programs of different challenges are grouped into different categories.

As presented in Fig. 3, programs of the same challenge are clustered into the same category and labeled with the same color. In contrast, programs of different challenges are grouped into different categories and labeled with different colors. The experimental result proves the previous conclusion that the vector embeddings generated by the proposed method contain all structural information on the original programs. Meanwhile, the vectors can effectively reflect the similarities and differences between the original programs.

#### Task definitions

To validate the HELP-DKT model, three different tasks, including Task A (next-challenge), Task B (next-attempt), and Task C (comprehensive analysis), are defined. All three tasks are designed for the purpose of verifying the improvement in the prediction accuracy and interpretability by using the proposed model.

- *Task A: Next-challenge* Based on all code vectors submitted by a student over time $T=[\mathbf{vec} _1, \mathbf{vec} _2, \ldots , \mathbf{vec} _k]$ for one programming challenge, the model predicts whether the student will successfully complete or fail the **next** challenge within specified number of attempts.
- *Task B: Next-attempt* At each time step $t\le k$, based on all previous code vectors submitted by a student before time step *t* (including time step *t*) over time *T* = [$\mathbf{vec} _1$, $\mathbf{vec} _2$, ..., $\mathbf{vec} _k$] for one programming challenge, the model predicts whether the student will successfully complete or fail the **current** challenge at time step ($t+1$).
- *Task C: Comprehensive Analysis* At each time step $t\le M$, where $M=\sum _{i=1}^{n} k_i$, $k_i$ represents the number of student’s attempts on challenge *i*, and *M* represents the total number of student’s attempts on all the challenges, based on *t* previous code vectors submitted by one student over time *T* = [$\mathbf{vec} _{11}$, $\mathbf{vec} _{12}$, ..., $\mathbf{vec} _{1k_1}$, $\mathbf{vec} _{21}$, ..., $\mathbf{vec} _{2k_2}$, ..., $\mathbf{vec} _{n1}$, ..., $\mathbf{vec} _{nk_n}$], where $\mathbf{vec} _{ij}$ represents the code vector on the *i*th challenge at the *j*th attempt, the model predict whether the student will successfully complete or fail the challenge at time step ($t+1$).

To sum up, Task A can be regarded as follows. For a given trajectory of a student’s practice of the previous challenge, it is predicted whether a student can learn new concepts of the next challenge. Further, Task B can be seen as providing real-time feedback to teachers since it predicts whether a student can complete the current challenge on the next attempt. Task C involves longer trajectories than Tasks A and B, so it is suitable for modeling students’ ability to implement all knowledge components while predicting the performance in the next attempt.

#### Implementation

The input vector and P-matrix are implemented to the LSTM network using the proposed methods. First, the 10-dimensional code vector is transformed into a 20-dimensional vector according to the *one-hot* encoding rule defined by Eqs. (4) and (5). It should be noted that the dimension of the code vector is equal to the number of programming concepts. Therefore, the code vector multiplied by the P-matrix is used as the input of the LSTM network.

Before training, parameters of the LSTM network are initialized to zeros, and parameters of the fully-connected layer are initialized uniformly in range (− 0.05, 0.05). All model parameters are optimized during the training process by minimizing the cross-entropy loss.

The model is trained using the Adam optimization with a learning rate of 0.01, a batch size of 32. Since all the input sequences are of different lengths, certain measures are conducted to ensure that sequences are of the same length. Namely, sequences a the length less than the maximum length are padded with zeros to fill up the remaining time steps. Also, masking is used in the loss computing process. The dataset is split on the student level, and the codes submitted by one student are all either in the training set or all in the test set. Thus, codes submitted by the same student do not repeatedly appear in the training and test sets. Eighty percent of the students’ codes are used as the training set, and the remaining 20% are used as the test set.

Specifically, task A predicts whether a student will successfully complete or fail the next challenge within a specified number of attempts. Therefore, a hyperparameter $try\_num$ is defined and set to three to decide on labels of sequences of task A. If the attempt number of the next new challenge is no more than $try\_num$, it is assumed the student will successfully complete the next new challenge, and the label of the current sequence is set to one (i.e., correct). In contrast, if the attempt number of the next challenge is larger than $try\_num$, the current sequence label is set to zero (i.e., failure).

## Results and discussion

### Prediction results

For the purpose of comparison, the DKT model and Deep-IRT model are used as baseline models and compared with the proposed model under the same dataset. All the three models are implemented using the PyTorch library on the same computer with four NVIDIA TESLA V100-SXM2 32GB GPUs. The experimental results are shown in Table 2. To compare the performances of the HELP-DKT and baseline models, five training and evaluation processes are conducted. In this study, the average and standard deviation of the area under the ROC curve (AUC) and the accuracy (ACC) are used as evaluation metrics. The larger the AUC or ACC score is, the better the model’s prediction performance is. The AUC is a robust overall measure that has been commonly used to evaluate the performance of binary classifiers because it avoids the supposed subjectivity in the threshold selection process.

**Table 2:** Prediction performances of the HELP-DKT, DKT, and Deep-IRT models.

	HELP-DKT		DKT		Deep-IRT	
	AUC	ACC	AUC	ACC	AUC	ACC
Task A	0.907 ± 0.007	0.853 ± 0.010	0.6862 ± 0.009	0.6448 ± 0.013	0.8344 ± 0.007	0.7898 ± 0.005
Task B	0.876 ± 0.011	0.875 ± 0.009	0.8661 ± 0.006	0.6950 ± 0.014	0.8061 ± 0.015	0.7069 ± 0.007
Task C	0.821 ± 0.019	0.863 ± 0.013	0.8160 ± 0.017	0.7087 ± 0.009	0.8172 ± 0.018	0.8058 ± 0.016

The results show that the proposed HELP-DKT model performs better than the DKT and Deep-IRT models in terms of the AUC and ACC indexes on each task. Such outstanding performance is attributed to the feature-rich input, including program vector embeddings and P-matrix. For Tasks A and B, the proposed model achieves higher prediction accuracy than for Task C. The main reason is that the input sequences of Task C are longer than those of Tasks A and B, which can increase the difficulty of correct prediction.

### Case study

**Figure 4:**
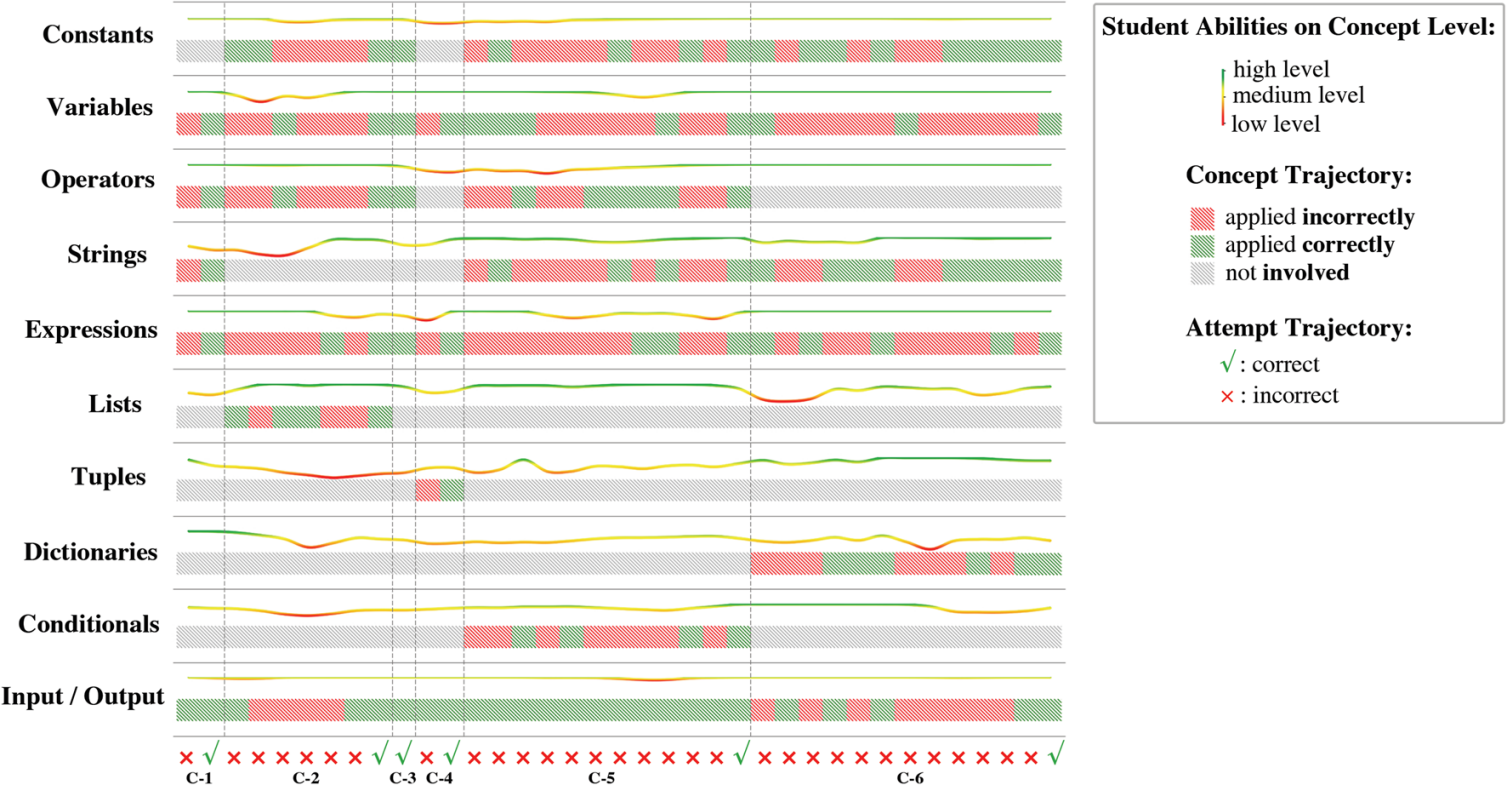
An example of a student’s submission trajectory from the dataset. The vertical axis shows 10 Python concepts, and the horizontal axis shows the attempt trajectory of the student over six challenges; $\times$ means the attempt is incorrect, while $\checkmark$ means that it is correct. In each concept area, student ability on a particular concept is presented by a curve in a different color. The curve in red means the student’s ability is at a low level, the yellow color indicates medium level, and the green color means the student masters the concept. Under the student ability curve, the blocks in red, green, and gray are used to show the student’s applying trajectory of each concept. The red block means the concept is applied incorrectly; the green one means the concept is applied correctly, and the gray one means this concept is not involved in the relevant challenges.

To demonstrate the HELP-DKT’s interepretability in analyzing dynamics of student’s abilities at the conceptual level, one student is randomly selected, and his abilities and learning trajectory of Task C are analyzed, as shown in Fig. 4. This student has completed all six challenges after a different number of attempts. The student has completed the challenges of “String concatenation” (C-1), “Modifying a list” (C-2), “Calculating quantities” (C-3), and “Sorting elements” (C-4) in a few attempts. Still, the student has struggled with the challenges of “Computing factorials” (C-5) and “Modifying a dictionary” (C-6), and has attempted to solve each of them at least ten times. Based on the inference of the HELP-DKT model, the temporal skill change at the conceptual level in the student’s long learning trajectory can be obtained.

As shown in Fig. 4, the prediction transition is smooth, and the changing trend of student ability is in line with the learning status on the whole. For instance, the ability level of the concept expressions is high for the first several time steps; the color of the curve is green, which indicates that the student masters this concept; thus, it is likely that the student listens carefully in class. However, the student’s ability starts to fluctuate after continuously failing in solving challenge C-2 in the first six attempts. When the student tries to answer challenge C-4 but does not use expressions to construct programming statements correctly, the ability curve of this concept turns to red, which indicates that the student does not handle expressions well. This can be because C-4 sorting algorithm demands higher mastery of the expressions concept. After continuing to tackle the challenge and finally succeeding to solve it, the student’s ability level shifts back to green color. Based on the results, the student has spent more attempts on challenges C-5 and C-6 than on the other challenges. Namely, as challenges become more complex, the student’s ability curve fluctuates more from the learning trajectory. Each time the student answers a challenge correctly, the ability curve of expressions increases. Clearly, the change in the student’s ability reflects whether the concept is applied correctly in solving the challenge. After completing all six challenges, the ability curves on each concept achieve levels that are higher than their initial levels, which confirms that the student has mastered all the concepts through the practice with the challenges.

By visualizing the trends of each student’s abilities at the conceptual level over time, rich information can be provided to instructors to analyze students’ mastery of key concepts and to identify common cognitive problems that occurred in programming exercises. The ability curves in Fig. 4 can enable course instructors to take personalized instructional intervene for novice learners and provide them with valuable feedback to help them to improve their skills in basic programming.

## Conclusions

In this study, a DKT-based cognitive model named the HELP-DKT intended for online programming courses is proposed. The proposed model adopts a rich-feature input layer by representing source codes of students’ submissions as vector embeddings and incorporates the error classifications as concept indicators into the personalized Q-matrix. Besides, the HELP-DKT introduces an additional cognitive layer in the basic DKT structure to infer accurate estimation of students’ abilities at the conceptual level and to present explainable temporal change in the conceptual skills of students. The proposed model is verified by experiments, and experimental results show that the proposed HELP-DKT model can achieve better interpretability and higher prediction performances than the DKT and Deep-IRT models. In future work, the HELP-DKT model will be evaluated using larger datasets, and the other state-of-art deep cognitive models will be explored beyond the DKT framework.

## Data Availability

The code and relevant dataset have been published in the following URL: https://github.com/liangyubuaa/HELP-DKT.
